# Metabolic changes in kidney stone disease

**DOI:** 10.3389/fimmu.2023.1142207

**Published:** 2023-05-09

**Authors:** Zhenzhen Xu, Xiangyang Yao, Chen Duan, Haoran Liu, Hua Xu

**Affiliations:** ^1^ Department of Urology, Zhongnan Hospital of Wuhan University, Wuhan, China; ^2^ Department of Urology, Tongji Hospital, Tongji Medical College, Huazhong University of Science and Technology, Wuhan, China; ^3^ Stanford Bio-X, Stanford University, San Francisco, CA, United States; ^4^ Department of Biological Repositories, Zhongnan Hospital of Wuhan University, Wuhan, China; ^5^ Cancer Precision Diagnosis and Treatment and Translational Medicine Hubei Engineering Research Center, Zhongnan Hospital of Wuhan University, Wuhan, China

**Keywords:** metabolism, calcium oxalate, ROS, macrophage, hormone, kidney stones

## Abstract

Kidney stone disease (KSD) is one of the earliest medical diseases known, but the mechanism of its formation and metabolic changes remain unclear. The formation of kidney stones is a extensive and complicated process, which is regulated by metabolic changes in various substances. In this manuscript, we summarized the progress of research on metabolic changes in kidney stone disease and discuss the valuable role of some new potential targets. We reviewed the influence of metabolism of some common substances on stone formation, such as the regulation of oxalate, the release of reactive oxygen species (ROS), macrophage polarization, the levels of hormones, and the alternation of other substances. New insights into changes in substance metabolism changes in kidney stone disease, as well as emerging research techniques, will provide new directions in the treatment of stones. Reviewing the great progress that has been made in this field will help to improve the understanding by urologists, nephrologists, and health care providers of the metabolic changes in kidney stone disease, and contribute to explore new metabolic targets for clinical therapy.

## 1 Introduction

Kidney stone disease (KSD), also known as urolithiasis or nephrolithiasis, is one of the most common urinary tract disorders (1). There is considerable evidence that the occurrence and prevalence of KSD have grown significantly in recent years (1-5% in Asia, 5-9% in Europe, and 7-13%in North America) (2, 3). Research on the morbidity of kidney stones in China showed that kidney stones affected 1 in 17 adults, and approximately two-thirds contain calcium oxalate (CaOx) (4). CaOx stones cause severe damage to renal function due to their high incidence, nearly 90% ten-year recurrence rate and long course of the disease, leading a great economic burden to patients and society (5, 6). Although great progress has been made in surgical therapy, the postoperative recurrence rate of CaOx calculi remains high, and the therapeutic effect of preventing recurrence of CaOx stones is not satisfactory. Previous studies have shown that gender, ethnicity, age, lifestyle and dietary habits are important factors during stone formation (7–9). Numerous recent studies demonstrate that the formation of kidney stones involves multiple metabolism-related factors, such as obesity, diabetes and metabolic syndrome (10–12), which are considered to be dangerous elements for stone formation, but the specific pathogenesis has not been elucidated.

Our previous research found that the most common urinary stone components can be divided into the following types: CaOx (65.9%), carbapatite (15.6%), urate (12.4%), struvite (2.7%), and brushite (1.7%). CaOx and urate stones were found to occur more frequently in males, while carbapatite and struvite were dominated in females. CaOx stones and carbapatite were mainly observed in people aged 30 to 50 years and 20 to 40 years. Brushite and struvite were most prevalent in those younger than 20 years and those older than 70 years. Carbapatite, brushite, CaOx and cystine stone were more common in the kidney than other types, while urate stone and struvite were formed in the bladder (13). According to whether they contain calcium, kidney stones are classified into calcium stones and noncalcium stones (14). Calcium-containing stones are the most general stone type, and a mixture of CaOx and calcium phosphate (CaP) accounts for nearly 80% of kidney stones (14, 15). Currently, the specific mechanism of 4 kidney stone formation remains unclear, and Randall’s plaque is considered the mainstream theory for the origin of CaOx stones. Initially, CaP crystals and organic substrates are deposited along the basement membranes of the thin loops of Henle and extend further into the interstitial compartment to the urothelium, forming Randall’s plaques (16). They make contact with the urine and become a core of tiny crystals that adhere to the urinary tract, and eventually develop into stones (17). Nevertheless, the 10 exact formation process of Randall's plaques still needs further perfection (18). Of course, stone formulation is a multistep chronic procedure involving crystal nucleation, crystal growth, crystal accumulation and crystal preservation (1, 15). Consequently, exploring the specific mechanism and valuable precaution for kidney stone development are the pivot issues to be solved in the future.

The pathophysiology of stone formation is complex, involving the effects of multiple metabolic changes, and the view of kidney stone formation is transforming from an isolated disease to a systemic disease (19). Accumulating evidence suggests that kidney stones are associated with systemic diseases such as obesity (20), diabetes mellitus (21), and cardiovascular disease (22). Moreover, patients with hypertension (23), bone disease (24) and metabolic syndrome (25) have a significantly increased risk of kidney stones, while kidney stone patients are often associated with chronic kidney disease (CKD) and kidney failure (26–28). Hyperoxalate, hypercalcium, and hyperuric acid metabolism are activated through multiple mechanisms, and these metabolic disturbances lead to supersaturation of urine to form CaOx/CaP crystals, which promote stone formation (29, 30). In addition, previous research has demonstrated that oxalate and crystal-induced inflammatory responses to renal tubular epithelial cells (RTECs) damage are closely associated with the pathological formation of kidney stones, and reactive oxygen-induced oxidative stress plays a key regulatory role (29). In general, mitochondrial damage is the classic origin of reactive oxygen species generation in renal epithelial cells stimulated by oxalic acid and/or CaOx crystals (31). Studies have found that nicotinamide adenine dinucleotide phosphate (NADPH) oxidase is involved in ROS production and kidney stone formation (32, 33). These studies further confirm the correlation between systemic metabolic diseases and kidney stones, and NADPH oxidase can be used as an emerging therapeutic target for kidney stones (34). Furthermore, David et al. showed that macrophage-associated inflammation and anti-inflammatory are major immune responses in the process of renal stones and involved in the formation of renal CaOx crystals (35). The crucial role of immune response in CaOx crystallization has led to the recognition that immunotherapy may provide a potential approach for preventing the recurrence of renal calculi in individuals by modulating the immune response (36).

We summarized and categorized the numerous researches related to kidney stones and metabolism over the past decade, and observed that researches on the role of metabolic factors in stone formation has received increasing attention (**Table 1**). This review provides an introduction to metabolic risk factors associated with the formation of KSD and discusses how metabolism-oriented therapies may be a prospective approach for the therapy and prevention of kidney stones.

**Table 1:** Relationship between metabolite changes and kidney stone formation.

PMID	Year	Author	Metabolin	Metabolic organs or cells	Pathway	Function
23833257	2013	Ohana et al.	Oxalate	/	SLC26A6	Regulate oxalate and induce stones formation
24956378	2014	Liang et al.	Oxalate/AR	/	NADPH oxidase-P22-phox	Regulate oxalate biosynthesis and oxidative stress
25175550	2014	Sasakumar et al.	oxalate	Intestine	/	Reduce oxalate excretion
27612997	2017	Mulay et al.	Oxalate	Kidney tubule	TNFR signaling pathway	Promote the adhesion of calcium oxalate crystals
29272854	2018	Patel et al.	Oxalate	/	/	Affect the mitochondrial function of monocyte
29395336	2018	Amin et al.	Oxalate	Intestine	A6mRNA/total protein	Reduce oxalate and stones formation
31117291	2019	Li et al.	COM/OA	/	NOX4/ROS/P38MAPK	Inhibit kidney stones formation
30894518	2019	Zhu et al.	Oxalate/AR	Kidney tubule	MiRNA-185-5p	Inhibit the phagocytosis of CaOx crystals
31475403	2019	Peerapen et al.	Oxalate/Estrogen	Kidney tubule	Annexin A1/α-enolase	Affect oxalate metabolism and prevent kidney stones
31178964	2019	Zhu et al.	Oxalate/ERβ	Liver	AGT1	Inhibit oxalate biosynthesis and prevent stones formation
34433051	2020	Gianmoena et al.	Oxalate/Glyoxylate	Liver	Downregulate hypermethylation and Agxt	Promote kidney stones formation and CKD
32317970	2020	Shimshilashvili et al.	Oxalate/Citrate	/	SLC26A6 - STAS domain structure	Affect citrate and oxalate homeostasis and induce CaOx stones
34783577	2021	Liu et al.	Oxalate/SCFA	Intestine	SLC26A6	Reduce urinary oxalate and renal CaOx stones
18804815	2008	Tsujihata et al.	ROS/ATO	Kidney tubule	/	Inhibit oxidative stress and kidney tubule injury
29843125	2018	Sun et al.	ROS/MUC4	Kidney tubule	ERK signaling pathway	Inhibit oxidative stress
29849862	2018	Qin et al.	ROS	Kidney tubule	NADPH oxidase/Ang II/AT1R	Reduce the expression of stone-related proteins
31408871	2019	Liu et al.	ROS	HK-2	P38MAPK/NADPH	Reduce the production of ROS and apoptosis of HK-2 cells
30599261	2019	Zhu et al.	ROS/DMF	/	Nrf2	Inhibit inflammation and regulate oxidative stress
31735555	2019	Liu et al.	ROS	/	HMGB1/TLR4/NF-κB	Promote kidney tubule injury
30995115	2019	Sugino et al.	ROS	Kidney	/	Inhibit kidney stones formation
31926579	2019	Li et al.	ROS	HK-2	APE up-regulation and redistribution	Stimulate reactive oxygen species and apoptosis in cells
32945480	2020	He et al.	ROS	/	GLUD1	Reduce oxidative stress
31733571	2020	Kang et al.	ROS/SOD	/	Autophagy-ERs response	Reduce kidney stones formation and prevent kidney
34512861	2021	Lv et al.	ROS/Oxalate	Kidney	NLRP3/Caspase-1/IL-1β	Induce inflammatory response and ROS production
34211634	2021	Wu et al.	ROS/Calcium	Kidney tubule	ROS/NF-κβ/MMP-9	Promote calcium crystal deposition
34506233	2021	Jia et al.	ROS/UA	Kidney	NRF2/HO-1/TLR4/NF-κβ	Inhibit oxidative stress
34204866	2021	Guzel et al.	ROS/Quercetin	Kidney	P38-MAPK	Reduce the damage caused by hyperoxaluria
33718378	2021	Wu et al.	ROS	Kidney	TFEB	Regulate inflammation and oxidative injury
24578130	2014	Taguchi et al.	M2	Kidney tubule	CSF-1	Inhibit renal crystal formation
30588609	2019	Xi et al.	M2/SIRT3	Kidney	FOXO1	Inhibit kidney calcium oxalate crystal formation
32641994	2020	Liu et al.	M2	/	Nrf2-miR-93-TLR4/IRF1	Inhibit inflammatory injury
11490302	2001	Yagisawa et al.	Androgen	Kidney	/	Promate kidney stones formation
27260493	2016	Changtong et al.	Androgen	Kidney	Increase α-enolase	Increase COM crystal cell adhesion
27857889	2016	Gupta et al.	Androgen	/	/	Enhance kidney stones formation
29953960	2018	Singhto et al.	Exosome	Macrophage	IL-8 production and neutrophil migration	Exosomes are involved in the inflammatory response in COM
29535716	2018	Singhto et al.	Exosome	Macrophage	/	Enhance the phagocytic activity of macrophages
31342142	2019	Sueksakit et al.	Androgen	/	/	Inhibit kidney stones formation
33845860	2019	Peng et al.	Androgen	kidney	HIF-1α/BNIP3	Induce renal tubular epithelial cells death
33852977	2021	Yuan et al.	AR/Kaempferol	Kidney tubule	AR/NOX2	Inhibit oxidative stress and inflammation
35500753	2022	Lee et al.	Estrogen	/	SLC26A6	Dysregulate oxalate transport
30548662	2018	Xi et al.	Sirtuin 3	/	NRF2/HO-1	Inhibit kidney stones formation
31639794	2019	Li et al.	VK-1	/	MGP	Inhibit renal crystals formation
34675921	2021	Jin et al.	SCFA	Kidney	CX3CR1CD24 macrophage	Inhibit calcium oxalate crystal formation
34606628	2021	Wei et al.	LPN1	Intestine	/	Prevent hyperoxaluria
34630369	2021	Liu et al.	Arginine	Intestine	/	Reduce renal CaOx crystals
33718066	2021	Hong et al.	Sodium	/	/	Increase the formation of stone
24948743	2014	Yu et al.	Calcium	Kidney tubule	Claudin 14	Mediate hypercalciuria pathogenesis
31449775	2019	Bouderlique et al.	Calcium/Vit-D	/	/	Accelerate Randall’s spots formation
32149733	2020	Curry et al.	Calcium	Kidney tubule	Claudin 2	Mediate calcium reabsorption
32197346	2020	Plain et al.	Calcium	Kidney tubule	Claudin 12	Mediate calcium reabsorption
25600098	2015	Whiteside et al.	Microbiome	Intestine	/	Diagnose and treat kidney stones
35524736	2022	Tian et al.	Microbiota	Intestine	TLR4/NF-κB/COX-2	Reduce kidney stones formation

AR, androgen receptor; A6mRNA, SLC26A6 mRNA; Agxt, alanine-glyoxylate aminotransferase; AGT1, glyoxylate aminotransferase 1; ATO, atorvastatin; Ang II, angiotensin II; AT1R, angiotensin receptor; APE, apurinic/apyrimidinic endonuclease; COM, calcium oxalate monohydrate; CaOx, calcium oxalate; CKD, chronic kidney disease; CSF -1, colony-stimulating; COX-2, Cyclooxygenase 2; DMF, dimethyl fumarate; ERβ, estrogen receptor β; ERK, extracellular signal-regulated kinase; ERS, endoplasmic reticulum stress; FOXO1, forkhead box O1; GLUD1, glutamate dehydrogenase 1; HK-2, human kidney epithelial cell line; HMGB1, high-mobility group box 1; IL1β, interleukin 1 beita; IRF1, interferon regulatory factor 1; IL-8, interleukin 8; LPN1, lactiplantibacillus plantarum N-1; MUC4, mucin 4; MMP-9, matrix metalloproteinase-9; M2, M2 macrophage; MGP, matrix Gla protein; NADPH, nicotinamide adenine dinucleotide phosphate; NOX4, nicotinamide adenine dinucleotide phosphate oxidase 4; Nrf2, nuclear factor (erythroid-derived 2)-like 2; NF-κB, nuclear factor kappa B; NLRP3, NOD-like receptor protein 3; NF-κβ, nuclear factor kappa-light-chain-enhancer of activated B cell; NOX2, nicotinamide adenine dinucleotide phosphate oxidase 2; OA, obcordata A; ROS, reactive oxygen species; STAS, sulfate transporter and anti-sigma factor antagonist; SCFA, short-chain fatty acid; SOD, Superoxide dismutase; Sirt3, sirtuin 3; SLC26A6, Solute Carrier Family 26 Member 6; TNFR, TNF receptor; TLR4, toll-like receptor 4; TFEB, transcription factor EB; UA, ursolic acid; VK-1, Vitamin K1; Vit-D, Vitamin D.

## 2 Oxalate metabolism

Oxalate is the main promoter of crystal formation and accelerates the crystallization or aggregation of stone components by activating several mechanisms (30). Therefore, oxalate metabolism in the human body seriously affect the formation of kidney stones (37). Oxalate homeostasis in humans is controlled by complex mechanisms, including epithelial transporters such as the oxalate transporter SLC26A6 (38). SLC26A6 is a conserved anion transporter that plays a critical role in ion homeostasis and acid-base balance (39). It is mainly exposed in the intestine and kidney and is engaged in the metabolism of oxalate *in vivo* as a major transporter of oxalate absorption and excretion (40). Short-chain fatty acids, a product of dietary fiber, decrease oxalate in urine and renal CaOx stones *via* SLC26A6 (41). Amin et al. demonstrated that proinflammatory cytokines (which were elevated in obese mice) obviously reduced oxalate transport in the intestine by reducing the level of SLC26A6(PMID:29395336). In addition, the SLC26A6-STAS domain can perceive and tightly regulate oxalate levels, interfere with oxalate homeostasis, and induce the formation of CaOx kidney stones (42). Moreover, studies have shown that many receptors participate in the crystal-cell interaction, which is considered to be the indispensable process for the retention of crystals in the kidney (43, 44). The calcium-sensing receptor (CaSR) is a G protein-coupled receptor that is activated by extracellular calcium and performs a wide range of functions. When stimulated by elevated serum calcium levels, it inhibits calcium reabsorption in the ascending limb and distal convoluted tubule and promotes CaP and oxalate precipitation. Therefore, CaSR can also prevent the precipitation of calcium oxalate in the urine by increasing the excretion of citric acid and water in the urine, thus playing a role in the formation of kidney stones (45–50).

Gianmoena et al. proposed that alanine-glyoxylate transaminase (Agxt) could detoxify glyoxylate and prevent excessive accumulation of oxalate. Downregulation and hypermethylation of Agxt were accompanied by an increase in oxalate production after the metabolism of precursor hydroxyproline. In contrast, Agxt is also downregulated and hypermethylated in hepatocytes from patients with nonalcoholic fatty liver disease (NAFLD), providing a mechanistic explanation for the increased risk of kidney stones and CKD in NAFLD patients (51). Another study indicated that *Bacillus subtilis* 168 (BS168) degrades oxalate and reduces the severity of calculi, also providing a new microbial therapy for stone treatment (52). Moreover, Li et al. indicated that Obcordata A (OA), a polyoxygestrel glycoside, prevents kidney stones through the mitogen-activated protein kinase (MAPK) pathway. Further studies revealed that OA regulates oxalate metabolism in RTECs by inhibiting nicotinamide adenine dinucleotide phospho oxidase 4 (NOX4) and downregulating NOX4/ROS/P38 proteins (53). In addition, therapeutic blockade of tumor necrosis factor receptor (TNFR) may provide a novel therapeutic approach for delaying oxalate nephropathy, as TNFR signaling is required for CaOx crystal adhesion to the lumen of the renal tubule, which is the basic initiation mechanism of oxalate nephropathy (54).

Ferroptosis is a nonapoptotic form of cell death, and numerous studies have indicated that ferroptosis is present in the pathophysiology of various diseases (55, 56). Oxalate metabolism has been found to induce autophagy and ferroptosis in human proximal tubules and promote the development and progression of kidney stones, which can be ameliorated by knocking down nuclear receptor coactivator 4 (NCOA4) (57). Patel et al. indicated that oxalate metabolism may have a close relationship with mitochondrial dysfunction in monocytes. Monocyte interactions with soluble and insoluble oxalates damage mitochondria and disrupt redox homeostasis (58). Moreover, proinflammatory cytokines dramatically reduced oxalate secretion and increased net oxalate absorption in the jejunum of active mice, clearly increasing the risk of hyperoxaluria and kidney stones (59). Consequently, inhibiting the expression and release of proinflammatory factors may be a potential strategy for stone control and prevention.

In addition, oxalate metabolism has been found to be regulated by endogenous hormones. Androgens increase the excretion of urinary oxalate, the concentration of plasma oxalate and the deposition of renal CaOx crystals. However, estrogen has the opposite effect (60). Liang et al. revealed that androgen receptor (AR) signal transduction contributes to the direct upregulation of glycolate oxidase in the liver and NADPH subunit P22-PHOx in the renal epithelium at the transcriptional level, which may upregulate oxalate biosynthesis. In contrast, targeting AR with the degradation enhancer ASC-J9 can inhibit this effect (61). Interestingly, Sueksakit et al. demonstrated that finasteride may be effective in preventing the testosterone-promoting role on kidney stone formation. It limits the conversion of testosterone to dihydrotestosterone, thereby inhibiting the effect of testosterone on oxalate metabolism. Furthermore, finasteride counteracts androgen-induced COM crystallization promotion as well (62). Conversely, estrogen acts as a protective factor for stones, but the exact mechanism is not clear. Related studies have revealed that there are two crystal receptors of CaOx on the plasma membrane, annexin A1 and α-enolase, both of which can enhance the crystal binding ability of renal tubular cells. Estrogen reduces the level of these two receptors and their crystal binding ability, thereby affecting oxalate metabolism and ultimately forming a protective effect on stones (63). Zhu et al. also confirmed in mouse experiments that estrogen receptor β (ERβ) inhibits oxalate biosynthesis and renal injury induced by oxidative stress through transcriptional upregulation of glyoxylate aminotransferase (AGT1) expression to prevent stone formation (64).

**Figure 1:**
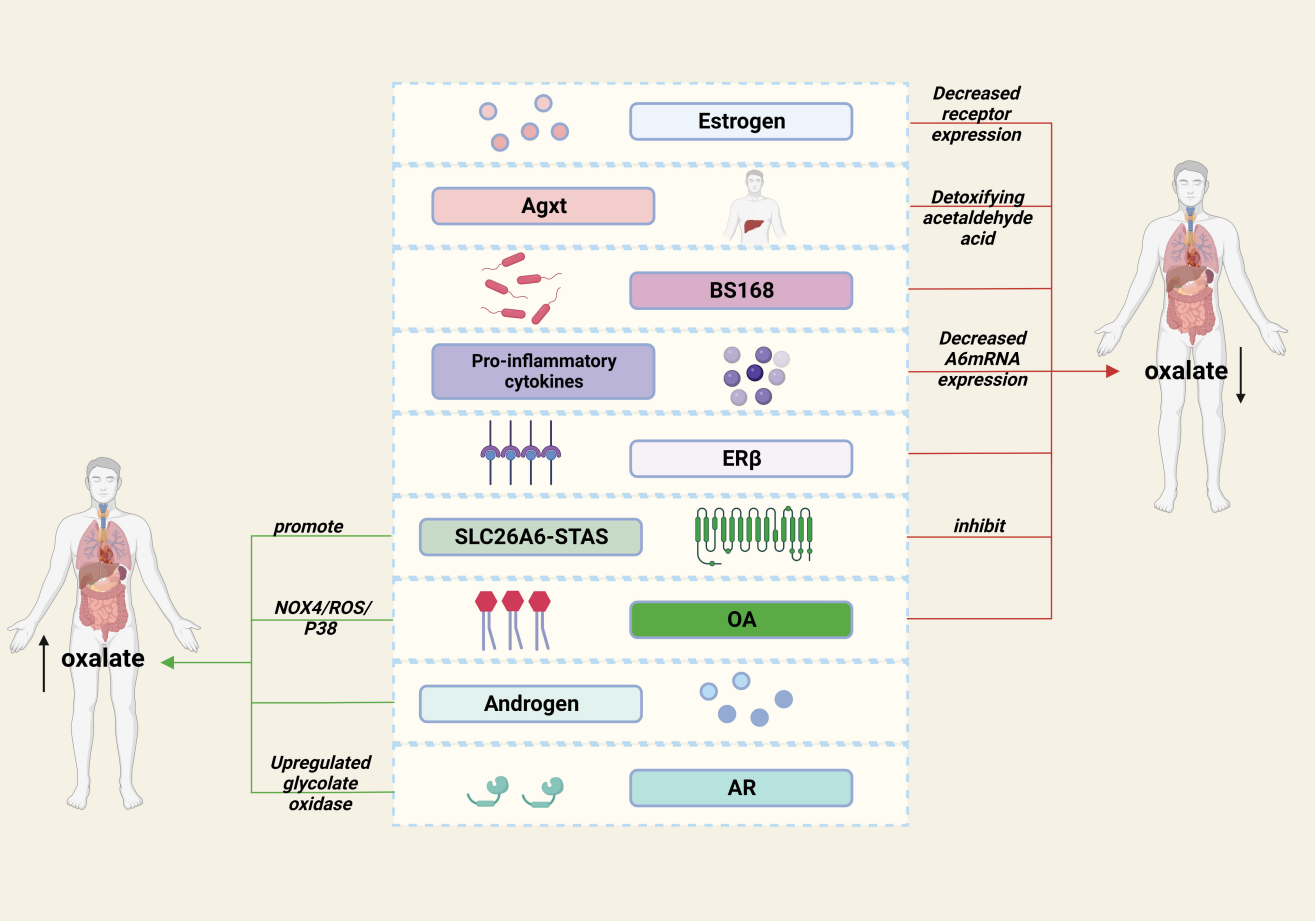
Factors affecting oxalate metabolism in kidney stones.

In conclusion, we review the substances that have been identified to affect oxalate metabolism in recent years (**Figure 1**), and we believe that there are three main ways to remove oxalate: reducing intake, increasing excretion and converting to other substances. Previous studies have shown that the intake of dietary oxalate, such as spinach, can promote stone formation (65, 66). Although it is not the main cause, it suggests that healthier dietary may prevent stones to some extent. In addition, increasing the transport, excretion and degradation of oxalic acid (SLC26A6, AR, ER, etc.) may be a valuable way, and the development of small molecular drugs targeting these molecules may attract more attention in the future. 

## 3 Metabolic changes in reactive oxygen species

Previous studies have revealed that the pathological formation of kidney stones is closely related to the injury and inflammatory response of RTECs, in which ROS-induced oxidative stress is indispensable (67, 68). As mentioned above, ROS mainly originates from injured mitochondria, while CaOx crystals significantly damage epithelial cell mitochondria and aggravate the inflammatory response (31). Excessive production of ROS by epithelial cells promotes crystal aggregation, growth, and adhesion, ultimately leading to stone formation (29). In addition, Khan et al. also suggested that ROS production and the progression of oxidative stress may be the common pathophysiological basis of kidney stones and other metabolic diseases (69).

**Figure 2:**
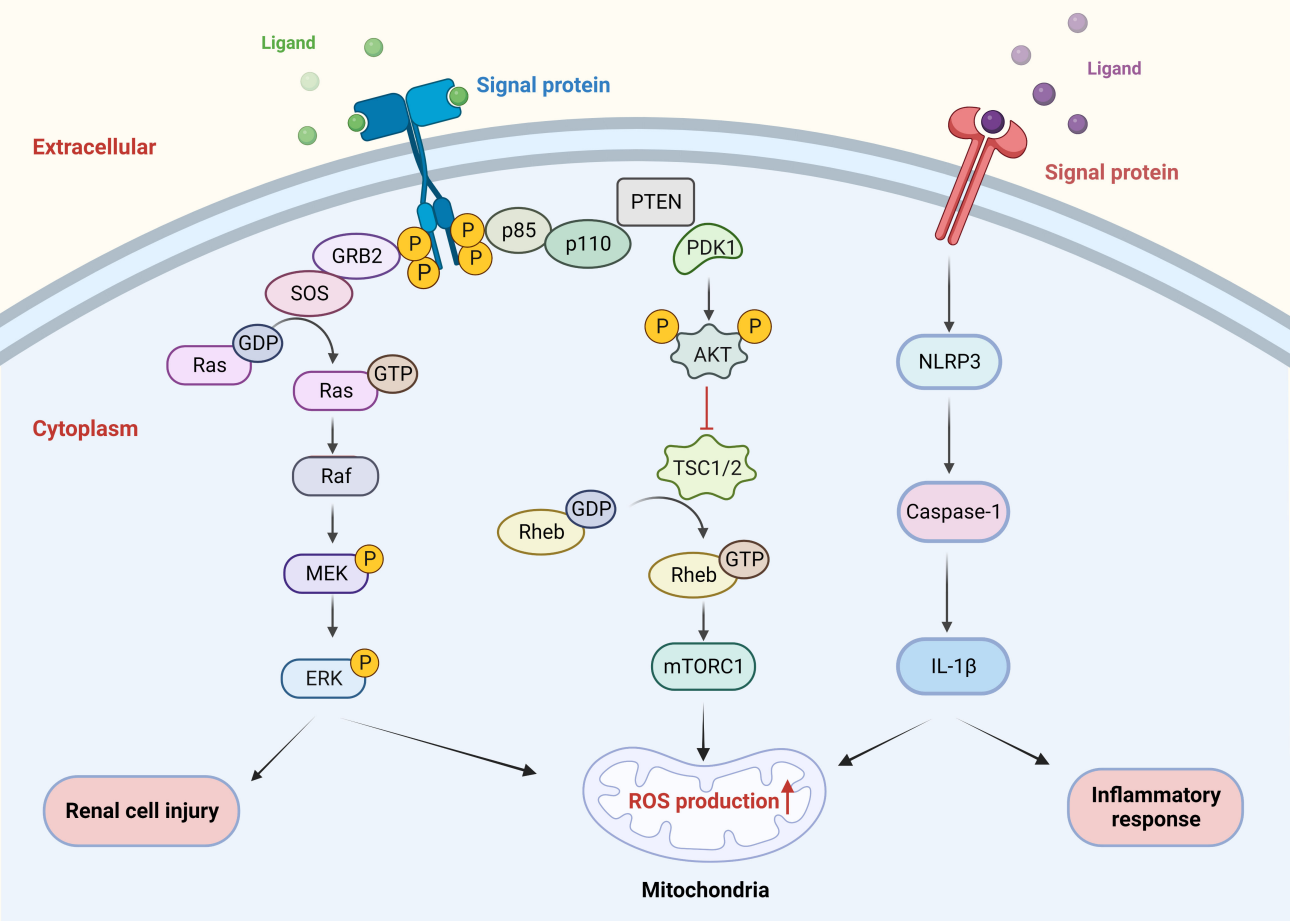
Signaling pathways involved in ROS. COM crystals induced renal cell injury by activating the ROS/AKT/P38 MAPK pathway. LncRNA XIST mediated the inflammatory response and ROS production through the NLRP3/caspase-1/IL-1β pathway interaction. ROS, reactive oxygen species; COM, calcium oxalate monohydrate.

Sirtuin (Sirt), a conserved family of proteins containing seven homologs (sirt 1-7), has nicotinamide adenine dinucleotide (NAD)-dependent protein deacetylase activity (70). Sirt3 is the major deacetylating enzyme in mitochondria and is indispensable in reducing ROS as well as ameliorating oxidative damage and mitochondrial dysfunction. Overexpression of sirt3 may activate the NRF2/HO-1 signaling pathway to reduce oxidative stress and apoptosis as well as the attachment of CaOx crystals to the surface of renal tubular epithelium (71). Accumulating evidences suggest that COM can induce the destruction of tight junctions and the injury of renal cells by activating the ROS/Akt/P38 MAPK signaling pathway, thereby enhancing the stone pathogenesis (72–74) (**Figure 2**). Li et al. showed that hyperoxalate induces ROS production apoptosis by aberrant expression, modification, and repartition of the apurate/pyrimidine endonuclide 1 (APE1) protein in HK-2 cells, which can be reversed by the antioxidant N-acetylcysteine (75). We have previously demonstrated the effect of lncRNA X inactive specific transcript (XIST) on ROS through mouse models. LncRNA XIST is involved in the formation and development of kidney stones by interactions with miR-223-3p and NLRP3/caspase-1/IL-1β pathways, mediating inflammatory responses and ROS production (76) (**Figure 2**). Enhanced superoxide dismutase (SOD) activity plays an important role in scavenging ROS by resisting oxidative stress (77). Tsujihata et al. proposed that atorvastatin (ATO) can inhibit kidney stones by enhancing SOD activity and is a new alternative therapy for preventing kidney stones (77–79). Since mucin 4 (MUC4) silencing inactivates ERK signaling pathways and further inhibits oxidative stress involving ROS in RTECs, it may also be a key target for stone prevention (80).

A high concentration of calcium is a stone promoter that accelerates the formation of kidney stones through the ROS/NF-κB/MMP-9 axis to promote epithelialosteoblast transition and calcium crystal deposition in renal tubules (81). Xun et al. showed that calcium could induce oxidative stress damage and apoptosis in RTECs through NOX4-derived ROS induced by PKC. In addition, calcium-mediated NOX4 abnormally activates bone morphogenetic protein 2 (BMP2) through MAPK signaling pathway, inducing renal tubular epithelial cells to transdifferentiate into osteoblasts and form renal calculi. This provides a new theoretical basis for the prevention and treatment of kidney stones (82). It is known that phospholipase A2(PLA2) can induce mitochondrial damage to produce excess ROS, while BS168 can blunt PLA2 to regulate ROS metabolism and thus affect stone formation (52). Increased calcium and/or oxalate in renal tubular solution may activate nicotinamide adenine dinucleotide phosphate(NADPH) oxidase in renal epithelial cells via the renin-angiotensin system and trigger the production of ROS(14978165 (83)). Other studies have found that M2 macrophages downregulate the activation of NADPH oxidase and reduce ROS production. Moreover, they can enhance the phosphorylation of Akt, inhibit the phosphorylation of P38 MAPK, and reduce oxidative stress damage and apoptosis of HK-2 cells (84). Consequently, abnormal activation of NADPH oxidase was confirmed to be involved in the formation of kidney stones (33). Dimethyl fumarate inhibits NADPH oxidase by modulating Nrf2, thereby affecting stone formation (85). Antihypertensive and lipid-regulating drugs such as candesartan, losartan and atorvastatin limit renal CaOx stones by suppressing NADPH oxidase-mediated ROS production (79, 86, 87). Atorvastatin also significantly inhibits oxidative stress and the TLR4/NF-κB and NLRP3 inflammasome pathways, ultimately improving CaOx crystal deposition and crystal-induced damage in HK-2 cells and renal tissue of rats (88). These studies have demonstrated the significant relationship between cardiovascular disease, metabolic syndrome, and kidney stones, which may have common targets in clinical treatment. Ursolic acid (UA) is a pentacyclic triterpene compound that has been used for centuries as an anti-inflammatory agent (89). Studies have shown that both high and low doses of UA can restore the levels of Nrf/HO-1 protein and SOD to reduce ROS production and effectively improve COM-induced oxidative damage and inflammation (90).

Hormones are important regulators of ROS production, and estrogen receptor β (ERβ) has been shown to inhibit oxalate-induced damage by reducing ROS production (64). Quercetin, a flavanol found in nuts, wine and vegetables, has strong antioxidant and immune activity (91). Guzel et al. demonstrated that quercetin not only is a strong scavenger of ROS but also enhances the total antioxidant capacity of plasma and reduces inflammation and oxidative stress in hyperoxaluria (92). Another nonflavonoid organic compound, resveratrol, has extensive anti-inflammatory and antioxidant effects (93). Resveratrol can significantly inhibit the production of ROS, reduce oxalate-induced oxidative stress, and play a protective role against kidney stones (94). MicroRNAs (miRNAs), as small noncoding RNAs, participate in multiple biological processes and modulate gene expression at the posttranscriptional level (95). He et al. suggested that mir-30a-5p could decrease ROS production in hypoxia/reoxygenation (H/R)-treated HK-2 cells by targeting glutamate dehydrogenase 1 (GLUD1) (96). In addition, as one of the most characterized long noncoding RNAs (lncRNAs), H19 is extensively engaged in the regulation of inflammation and induction of tissue damage. Previous studies by our group indicated that the activation of H19 can induce ROS outburst and renal tubular cell injury, further promoting CaOx crystal deposition in the kidney (97). Consequently, targeting noncoding RNAs may be a novel therapeutic strategy for renal injury.

Urolithiasis is a multifactorial and multistep metabolic disease. The metabolic changes of ROS affect the changes of oxidative stress, which plays an essential role in the formation of urolithiasis. Therefore, targeting ROS induced oxidative stress provides us with a new direction for stone prevention (98).

## 4 Changes in polarization and metabolism of macrophages

**Figure 3:**
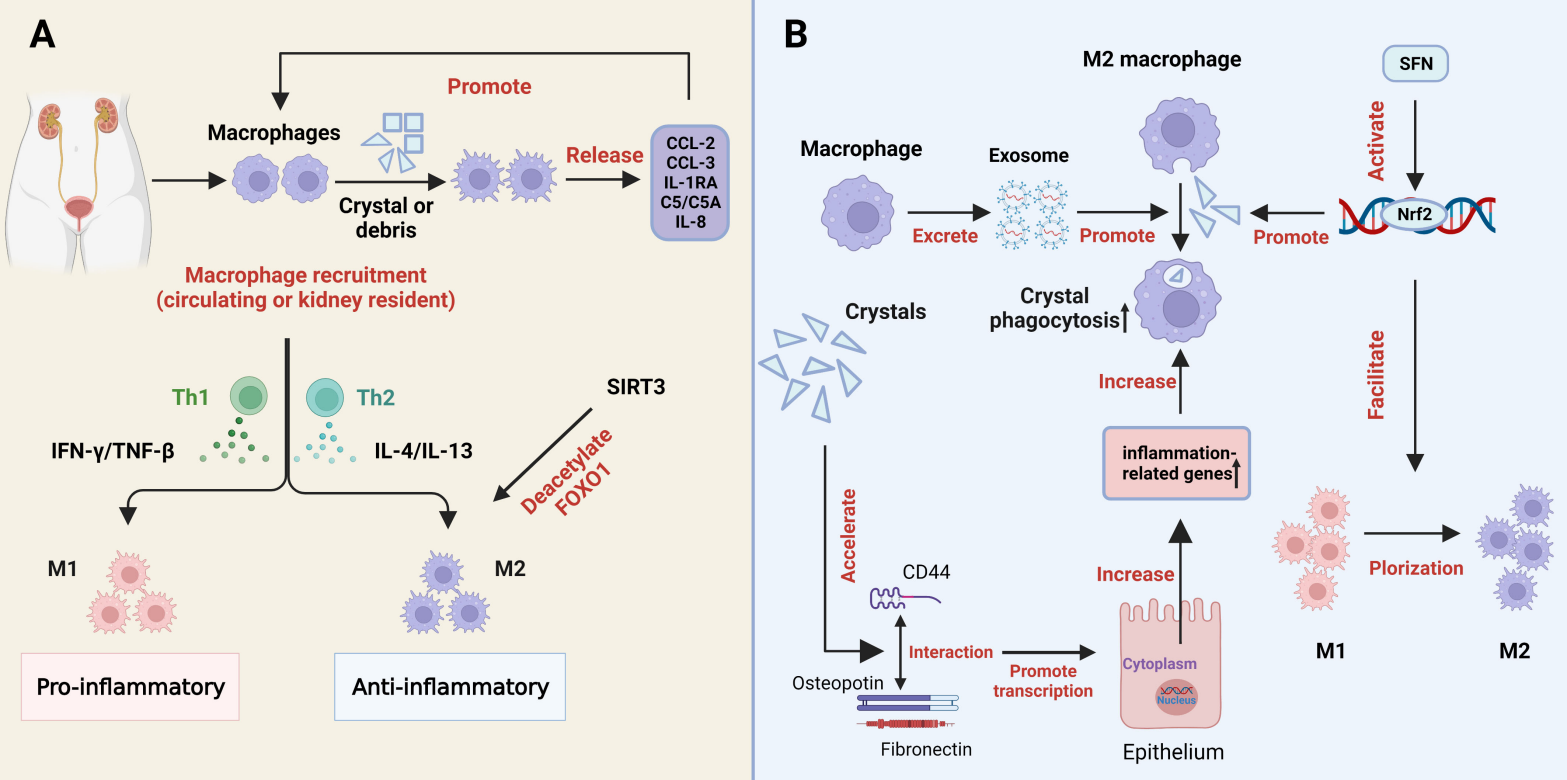
The role of macrophages in kidney stone formation. **(A)** Processes and regulators in the differentiation of macrophages into pro-inflammatory and anti-inflammatory macrophages. **(B)** Crystals can promote the expression of inflammation-related genes in renal tubular epithelial cells by accelerating the interaction of CD44 with osteopotin and fibronectin, thereby increasing phagocytosis by macrophages. Macrophage-derived exosomes increase the phagocytic activity of macrophages. SFN promotes the polarization of M1 macrophages to M2 macrophages and the phagocytosis of M2 macrophages through the activation of Nrf2. COM, calcium oxalate monohydrate; SFN, Sulforaphane; M1, M1macrophage; M2, M2 macrophage. SIRT3, sirtuin3.

Macrophage accumulation and macrophage-associated inflammation or anti-inflammatory effects have been widely reported in renal stone disease (35). The process of macrophages polarization is also a metabolic alteration which regulate the metabolism of stones in two forms (the pro-inflammatory M1 and the anti-inflammatory M2 in metabolic processes) (99, 100). Interferon γ (IFN-γ) and tumor necrosis factor-β (TNF-β) from Th1 cells tilted the polarization toward M1, while IL-4 and IL-13 secreted by Th2 cells promoted M2 polarization, which depends on the amount of cytokines, exposure time, and cytokine competition (101). M1Mφs promote the development of renal crystals, while M2Mφs reduce the expression of proinflammatory factors and suppress the development of stones through crystal phagocytosis (102) (**Figure 3A**).

Previous studies have shown that macrophages recognize and are activated by CaOx crystals through specific mechanisms, while CaOx crystals promote the differentiation of M1 macrophages and the production of inflammatory cytokines (103). Macrophage uptake of kidney stone debris or crystals triggers the release of typical cytokines, including chemokine CCL2 (monocyte chemotactic protein-1 and MCP-1), CCL3 (MIP-1α), interleukin-1 receptor antagonist (IL-1RA), complement components (C5/C5a), and IL-8 (CXCL-8). These factors can in turn promote macrophage recruitment (104) (**Figure 3A**). We recently found that metformin could attenuate crystal deposition and kidney injury by promoting sirt1 activation and M2Mφs polarization. Strong evidence is provided to support the therapeutic and preventive potential of metformin use in the clinic, especially in stones patients with diabetes (105). Xi et al. explored that sirt3 was able to deacetylate FOXO1 to promote macrophage polarization to the M2 phenotype (**Figure 3A**), and inhibit stone formation. The levels of sirt3 in peripheral blood mononuclear cells of patients with stones were significantly reduced, as was also demonstrated by elevated levels of acetylated FOXO1 (106). Zhu et al. also proposed another approach to promote M2 polarization and macrophage recruitment for stone treatment, through the expression of ACJ-9 targeting AR (107). In addition, our previous study found that sulforaphane (SFN), a pharmacological promoter of nuclear factor-erythroid 2-related factor 2 (Nrf2), facilitates M2Mφ polarization and phagocytic ability, thereby inhibiting CaOx-induced epithelial cell damage (108) (**Figure 3B**). Through gene sequence analysis, Taguchi et al. reported that the deficiency of colony-stimulating Factor 1 (CSF-1) may lead to the dysregulation of M2 macrophages and stone-related gene expression, suggesting that the CSF-1 signaling pathway acts as an inhibitor in renal crystal formation (109). Interestingly, exosomes, single-membrane secretory organelles rich in nucleic acid, protein, and lipid complexes, have been found to be involved in inflammatory and immune responses (110, 111). Singhto et al. indicated that macrophage exosomes are critical for the inflammatory response in COM crystals, enhancing the migration of monocytes and T cells as well as the phagocytic activity of macrophages (112) (**Figure 3B**). Meanwhile, they enhanced the production of the proinflammatory cytokine IL-8 by monocytes and increased the fragility of the exosome membrane and the binding ability to COM (112, 113). Furthermore, exosomes from CaOx-treated macrophages facilitate apoptosis of HK-2 cells through increased autophagy, suggesting that they may be involved in CaOx-induced renal tubular injury (114). In addition, COM crystal-induced increased enolase-1 secretion affects renal interstitial crystal invasion and inflammation (115). Therefore, cytokines play significant physiological roles in the prevention of kidney stone disease. Inhibiting the effect of exosomes may also become a direction for inhibiting stone formation in the future.

Crystal deposition promotes the interaction of CD44 with osteopontin and fibronectin, thereby accelerating the expression of inflammation-related genes in renal tubular cells, which increases the phagocytosis of monocyte-macrophages (**Figure 3B**). Therefore, this decrystallizing ability of macrophages may be a new target for the prevention of stone formation (116). Jin et al. revealed that a strong correlation exists between short-chain fatty acids (SCFAs), immune cells, and kidneys during the formation of calcium oxalate stones. SCFAs inhibit kidney CaOx crystal formation by regulating macrophage function in a microbiota- and GPR43-dependent manner (117). Moreover, SCFAs are not only significantly associated with antioxidant and mitochondrial stress, but also play a critical role in energy metabolism, insulin resistance and colonic function, and they may be a potential clinical therapeutic target (118–121).

Polarization is not fixed at different stages of different tissues and is regulated by multiple factors. The epigenetic regulation that affects the survival and development of macrophages, the tissue microenvironment, and the pathogenic microorganisms and cytokines in inflammation are the principal approaches to regulating the polarization of macrophages (101). CaOx crystals regulate macrophage polarization and phagocytosis in various ways, and activated macrophages further aggravate the deposition of crystals and the progression of kidney stones. Due to the powerful role of macrophages in calcium oxalate formation, methods of preventing stone recurrence in individuals by immunotherapy have been proposed (36).

## 5 The role of hormone metabolism

**Figure 4:**
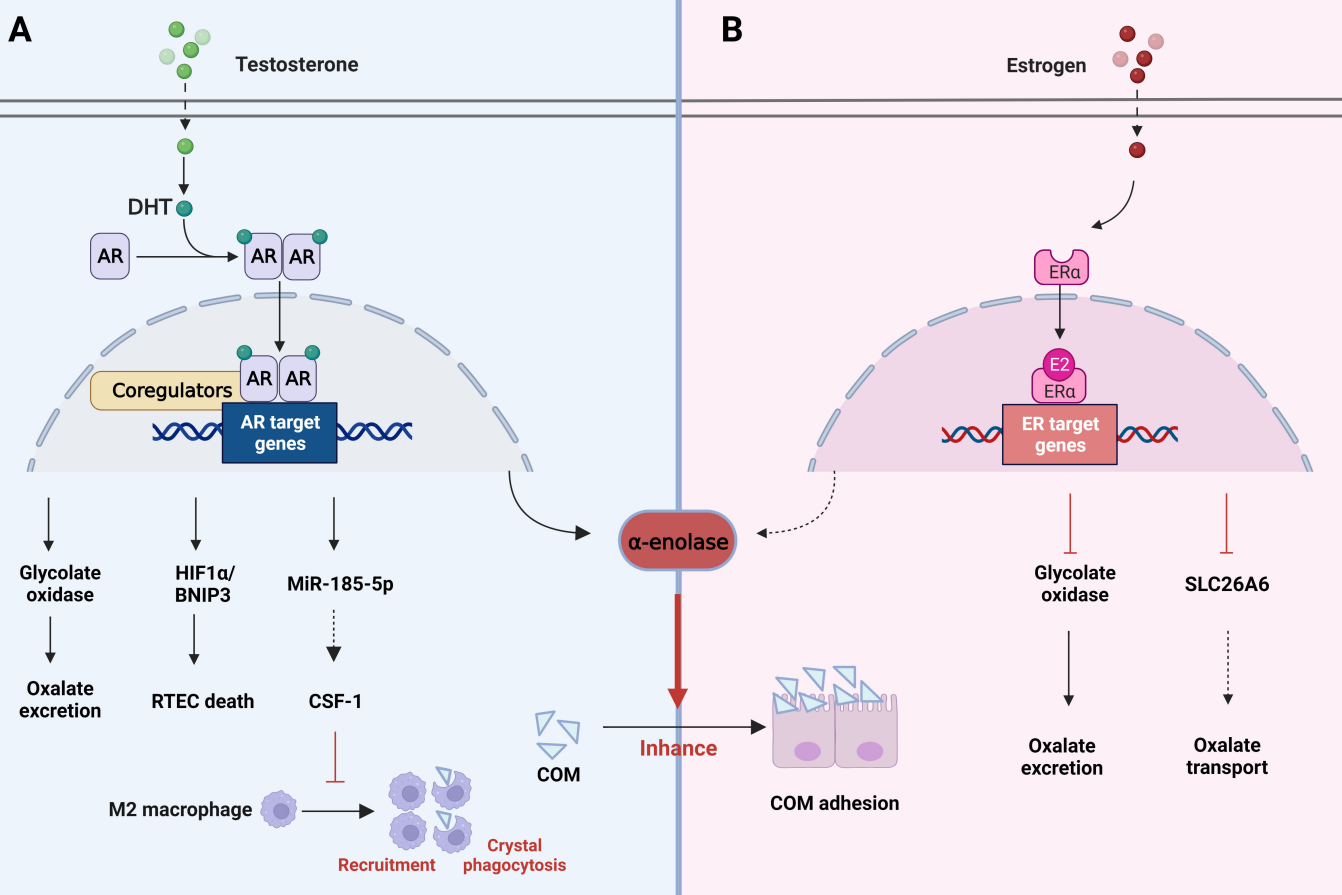
Role of changes in hormone metabolism in kidney stones. **(A)** AR signaling can induce the death of renal tubular epithelial cells by activating the HIF-1α/BNIP3 pathway, promote oxalate excretion and COM adhesion through glycolate oxidase and a-enolase. However, it decreases macrophage CSF-1 by increasing the expression of miRNA-185-5P, thereby inhibiting macrophage recruitment and crystal phagocytosis. **(B)** ER signaling affects oxalate metabolism by inhibiting glycolate oxidase activity as well as SLC26A6 transporters. Meanwhile, it also reduces a-enolase to reduce COM adhesion. AR, androgen receptor; COM, calcium oxalate monohydrate; ER estrogen receptor.

According to statistical analyses, a significant difference was found in the prevalence of stones between men and women, suggesting that hormone metabolism may exert an essential regulatory role in the formation of stones (7, 60). Earlier studies have shown that androgen appears to inhibit bone bridge protein levels and increase urinary oxalate excretion, whereas estrogen exhibits the opposite effect. In addition, androgen leads to increased synthesis of glycolate oxidase and excretion of urinary oxalate, which causes a higher incidence of urinary CaOx stone formation (122). In addition to the direct effects, sex hormones indirectly affect renal calcium excretion by regulating intestinal or bone calcium metabolism (123). These findings may partly explain the greater prevalence of stones in men. Yoshihara et al. noted a sex difference in the conversion of glycolate to oxalate in rats. Androgen promotes glycolate oxidase and affect urine oxalate excretion, while estrogen reduces glycolate oxidase activity (124) (**Figure 4A**). In addition, androgen appears to accelerate stone formation through suppression of osteopontin in the kidney and augmentation of urinary oxalate excretion. Nevertheless, estrogen has the opposite effect (125). The adhesion of COM crystals to the apical surface of RTECs is the early stage of kidney stone formation (126, 127). Androgen increases surface α-enolase (which acts as an enhancer of COM crystal binding (128)) to enhance the adhesion of COM crystals to the apical membrane of RTECs. Therefore, blocking cell adhesion may be an effective method for preventing stones (129, 130). Furthermore, androgen induces RTECs death by activating the HIF-1α/BNIP3 pathway, and death of RTECs is an important pathophysiological process leading to the development of kidney stones (131) (**Figure 4A**).

A study found that augmented androgen receptor (AR) signaling may be responsible for the link between hormones and kidney stones. AR signaling directly upregulates glycolate oxidase in the liver and NADPH oxidase subunit P22-PHOx in the renal epithelium. This upregulation may enhance oxalate biosynthesis and oxidative stress, resulting in stone formation (61). In addition, Zhu et al. found that suppression of the AR level in RTECs promotes macrophage recruitment, leading to enhanced intrarenal CaOx crystal phagocytosis. A mechanistic analysis demonstrated that AR could decrease macrophage CSF-1 by increasing miRNA-185-5p expression, inhibiting the phagocytosis of CaOx crystals mediated by M2 macrophage polarization (**Figure 4A**). Yuan et al. showed in their study that kaempferol (one of the most common flavonoids) could inhibit AR expression, oxidative stress and inflammation by regulating the AR/NOX2 signaling pathway. Meanwhile, CaOx crystal deposition and crystal-induced kidney damage were reduced, and stone formation could be inhibited as well (132).

In contrast, estrogen is known to decrease membrane fibronectin and α-enolase (**Figure 4B**), which are increased by high calcium and oxalate, thereby preventing stone formation. ERβ inhibits oxalate biosynthesis and the resulting damage, all of which provide a direction for the prevention of stone formation. β-estradiol treatment has also been shown to inhibit SLC26A6 activity to disrupt oxalate transport (133) (**Figure 4B**). These studies indicate that hormone metabolism has a significant influence on the formation of kidney stones, which provides us with a new research idea for the prevention of stones. Finasteran, a 5α reductase inhibitor, has been proposed for the treatment of kidney stones, but its specific efficacy and side effects need further validation and clinical studies. ERβ and MUC4 have been found as targets for stone prevention, but more potential targets need to be further explored.

## 6 Other metabolic changes

The process of kidney stones is regulated by multiple factors, in addition to the elements we have mentioned above, the metabolism of many other substances is indispensable. *Lactobacillus* (LAB) is involved in the metabolism of intestinal microorganisms, degrading oxalic acid *in vitro*, and reducing uric acid and kidney stones *in vivo*. A study of South African men found that Lactobacillus, which degrades oxalate, was associated with a lower risk of calcium oxalate kidney stones (134). Liu et al. isolated a *Lactobacillus plantarum* N-1 (LPN1) strain from traditional cheese that regulates arginine metabolism in the intestinal microbiome to reduce CaOx crystals in the kidney (135). LPN1 reduced oxaluria, renal osteopontin and CD44 expression by enhancing intestinal barrier function, ultimately inhibiting renal crystal deposition. It also ameliorated ethylene glycol (EG)-induced intestinal inflammation and barrier function by reducing serum LPS and TLR4/NF-κB signaling, upregulating tight junction claudin-2 and enhancing the production of short-chain fatty acids (SCFAs) in the colon (136). In addition, Tian et al. indicated that *Lactobacillus plantarum* J-15 may reduce kidney stones by restoring intestinal microbiota and metabolic disorders, protecting intestinal barrier function and alleviating inflammation (137). Sasikumar et al. directly demonstrated that artificially colonized *Lactobacillus plantarum* could increase intestinal oxalate metabolism, reduce urinary oxalate excretion and crystal deposition in mice (138). All these findings suggest that lactobacillus-containing probiotics may be a potential therapy for stone prevention, providing new insights into the prevention of kidney stones. Previous studies have shown that cystinuria can cause kidney stones and obstruction, whereas new drugs including alpha-lipoic acid may reduce stone deposition by accelerated the urinary solubility of cystine without affecting the recovery of cystine transport (139). This therapy may reduce stone development but probably not improve cystine or oxidative metabolism (140). Other studies have revealed that a low-protein diet and a high intake of plant protein can reduce the excretion of cystine, thereby affecting stone formation (140, 141).

Most kidney stones contain CaOx, many of which are derived from Randall’s plaques. Vitamin D increases urinary calcium excretion, which presumably hastens the formation of Randall’s plaque and kidney stones. In some genetically susceptible individuals, the combination of calcium and vitamins may also accelerate the formation of Randall’s plaque (142). Similarly, vitamin K1 (VK1) metabolism affects kidney stones through increased matrix glass protein (MGP) expression and function, reducing intracellular crystal deposition and providing cytoprotection. Therefore, VK1 therapy may be a potential tactic for the cure and prevention of kidney stones (143). Understanding the relationship between vitamin metabolism and stone formation also provides us with a new research direction.

A high or low sodium concentration is a regulator of metabolic changes in patients with kidney stones. Sakhaee et al. found that high sodium intake increased calcium excretion and urinary pH and decreased citric acid excretion (144, 145). In addition, high sodium intake may reduce calcium reabsorption by the renal tubules, resulting in increased urinary calcium and stone formation (146). According to Emamiyan et al., chicory flower extract reduced urinary oxalic acid levels and increased urinary calcium and creatinine metabolism at high doses, which may be related to the prevention of kidney stone formation. However, the exact mechanism of low dose on calcium stones needs further study (147). Claudin-2 is a cation-selective protein located on the epithelial cells of renal tubules that can affect calcium reabsorption, metabolism and stone formation (148, 149). In addition, claudin-12, another protein on RTECs, is also involved in calcium permeability (150). Claudin-14 is considered to induce the pathogenesis of hypercalciuria and kidney stones (151). Therefore, claudin-2, claudin-12 and claudin-14 may be potential targets for the prevention of kidney stones. In accordance with prior studies, kidney stones are related to metabolic syndrome. Sugino et al. found that β3-stimulant-induced brown-like adipocytes reduce the metabolism of renal inflammation and improve antioxidant effects, which in concert inhibit the formation of renal crystals (152).

Microorganisms in the kidney and urinary tract may have important implications for urinary tract health as a result of their metabolic regulation and contribution (153). Urease-producing bacteria, such as *Aspergillus chimaera*, *Klebsiella pneumoniae*, *Staphylococcus aureus*, *Pseudomonas aeruginosa*, *Providence, Siala, Mr. Charest’s* and *Morgan’s bacteria*, regulate calcium phosphate formation by degrading urea and promoting the production of carbon dioxide, thus playing a role in stone formation (154, 155). Gao et al. proposed that *E. coli, Staphylococcus*, and *Lactobacillus* were strong predictors of renal calculi and first reported that *Mycoplasma* and *Micrococcus* were also involved in kidney stones, but their potential significance in kidney stones still needs to be studied in more detail (156).

## 7 Conclusions

Metabolism-related factors, which have received considerable attention over the past decade as modulators of kidney stones, are discussed in this brief review. Although our functional understanding of the different metabolic members is still at an early stage, their active roles in stone regulation and disease treatment have become potential targets for future clinical researches. Oxalate is the main component of the most common stones known to date, and the mechanism by which oxalate regulates stone formation has been extensively studied. However, our team has previously found that recombinant lactic acid bacteria expressing oxalate degrading enzymes can be used for the oral treatment of hyperoxaluria (157). This provides us with a direction that dietary and pharmacogenic degradation or reduction of oxalate levels may be an effective measure to prevent kidney stones. ROS-induced oxidative stress damage plays an important role in crystal invasion of renal tubular epithelial cells. Although a large number of substances and signaling pathways have been reported to be involved in the inhibition of ROS production, there is still a need to discover additional pathways to inhibit mitochondrial damage due to its ROS production role. Previously, metabolic alterations in kidney stones were considered to the metabolism of certain substances. However, we believe that metabolic changes in kidney stone disease cannot be narrowly defined as metabolic alterations of several specific substances and should include the whole relevant metabolic activities involved in the process. Therefore, how to promote the transition of macrophages to M2 and inhibit the polarization of macrophages to M1 will be the focus of future research. The regulation of hormone metabolism on kidney stone formation explains the difference in stone prevalence between men and women. Postmenopausal women are more likely to develop stones due to a drop in estrogen levels (13). Therefore, estrogen supplementation may be a potential target for preventing stone formation and progression in the future.

By summarizing the effects of oxalate, ROS, macrophages, hormones and other substances on the formation of kidney stones, this article reviews the metabolic risk elements associated with KSD and provides an overview of the metabolic substances that promote and inhibit kidney stone formation. KSD is increasingly recognized as a multifactorial metabolism-related disorder rather than an isolated disorder. However, due to the limitations of current studies, the metabolic changes in stone formation are not well understood. Therefore, future studies are needed to further clarify the metabolic changes associated with kidney stone formation and develop new prevention and treatment strategies.

## Data availability statement

The original contributions presented in the study are included in the article/supplementary material. Further inquiries can be directed to the corresponding author.

## Author contributions

ZX and XY contributed equally to this work and are accountable for the content of this work. CD and HL revised the paper and provided some recommendations. HX conducted the final manuscript editing. All authors contributed to the article and approved the submitted version.
